# Response to Letter to the Editor “Keeping Late Thrombectomy Imaging Protocols Simple to Avoid Analysis Paralysis”

**DOI:** 10.1007/s00062-021-01091-5

**Published:** 2021-09-07

**Authors:** Franziska Dorn, Moriz Herzberg

**Affiliations:** 1 Department of Neuroradiology, University Hospital Bonn, 53127 Bonn, Germany; 2 Department of Diagnostic and Interventional Radiology, University Hospital Würzburg, 97080 Würzburg, Germany

Dear Johanna and Mayank,

Thank you very much for your positive feedback and valuable comments and thoughts on our paper [1].

We do agree that poorer clinical results compared to randomized trials should not frustrate us but rather seize the opportunity to learn from real-world data. DAWN and DEFUSE‑3 were necessary to prove the efficacy of endovascular treatment (EVT) in a late time window and opened the treatment to another large group of patients; in order to do so, the most promising patient population had to be selected [2, 3]; however, the low number needed to treat (NNT 2–4) in both the DAWN and DEFUSE- 3 trials already indicates room for broader selection criteria. We found that the majority of the patients in our multicenter real-world cohort in a late time window did not meet the DAWN/DEFUSE‑3 criteria, mainly because computed tomography (CT) perfusion was not performed or may not have been available [4]. Interestingly, the patients in our study who were treated without CT perfusion tended to do better than the patients who were selected by CT perfusion imaging.

It is certainly time to question the value of CT perfusion imaging for selecting patients for EVT and our data contribute to this question. CT perfusion may steal valuable time and may also lead to decisions against rather than in favor of treatment [5]. We already learned from ESCAPE-NA1 that patient selection based on Alberta Stroke Program Early CT Score (ASPECTS) and multiphase CT angiography (mCTA) had similar clinical outcomes when compared with patient selection based on CT perfusion imaging [6]. The MR CLEAN LATE study will provide us with more information on patient selection by visualization of collaterals in a late time window [7]. Until a reliable algorithm is defined, the majority of the German Stroke Registry centers would be well-advised to continue even without CT perfusion: in case of doubt, consider treatment.
